# Supplementary material for: The Spatiotemporal Expression of Notch1 and Numb and Their Functional Interaction during Cardiac Morphogenesis
Source: Cells. 2021 Aug 25;10(9):2192. doi: 10.3390/cells10092192 (PMC8471136; doi:10.3390/cells10092192)
Supplement: Supplementary file 1 [file cells-10-02192-s001.zip › cells-1340687-supplementary.pdf]

The Spatiotemporal Expression of Notch1 and Numb and Their Functional Interaction during Cardiac Morphogenesis

Lianjie Miao [1], Yangyang Lu [1], Anika Nusrat [1], Hala Y. Abdelnasser [1], Sayantap Datta [1], Bin Zhou [2], Robert J. Schwartz [3] and Mingfu Wu [1,*]

[1] Department of Pharmacological and Pharmaceutical Sciences, College of Pharmacy, University of Houston, Houston, TX 77204-5039, USA; lmiao4@central.uh.edu (L.M.); ylu37@central.uh.edu (Y.L.); anusrat2@CougarNet.UH.EDU (A.N.); habdelna@CougarNet.UH.EDU (H.Y.A.); sdatta20@CougarNet.UH.EDU (S.D.)

[2] Department of Genetics, Albert Einstein College of Medicine of Yeshiva University, Bronx, New York, NY 10461, USA; bin.zhou@einsteinmed.org

[3] Department of Biology and Biochemistry, University of Houston, Houston, TX 77204-5001, USA; sjrobert@central.uh.edu

* Correspondence: mwu25@central.uh.edu; Tel.: +713-743-9880

Supplemental materials and methods

mRNA deep sequencing

mRNA deep sequencing was performed as previous described. Briefly, total RNA was isolated with Aurum Total RNA Mini Kit (Bio-Rad, 732-6820) from three E11.5 whole hearts from control, MDKO and TKO hearts for each experiment. As an indication of quality, the RNA had an integrity number of 8 or greater by Bioanalyzer (Agilent Technology). Samples for mRNA deep sequencing were prepared according to the manufacturer's protocol (mRNASeq 8-Sample Prep Kit, Illumina). The samples were sequenced by the Microarray Core Facility at the University of Texas, Southwestern Medical Center at Dallas. A HiSeq 2000 system (Illumina) was used for SE-50 sequencing (single-ended 50 bp reads), with over 30×10^6 'reads' per sample. Basic data analysis was performed with CLC-Biosystems Genomic Workbench analysis programs to generate quantitative data for all genes. The quality filtered and trimmed reads were aligned to an annotated mouse reference genome downloaded from the Ensembl Genome Browser. cDNA fragments were mapped back to individual transcripts. After normalization, the RNA-Seq fragment count was used as a measure of relative abundance of transcripts, and CLC BIO measured transcript abundances in reads per kilobase of transcript per million mapped reads (RPKM). The experiment was repeated three times. Relative expression levels of genes (ratio of TKO to MDKO) with $P<0.05$ were considered significantly different. Genes with expressional levels are up-regulated or down-regulated two times are used to perform Gene Ontology assay using String DB (https://string-db.org).

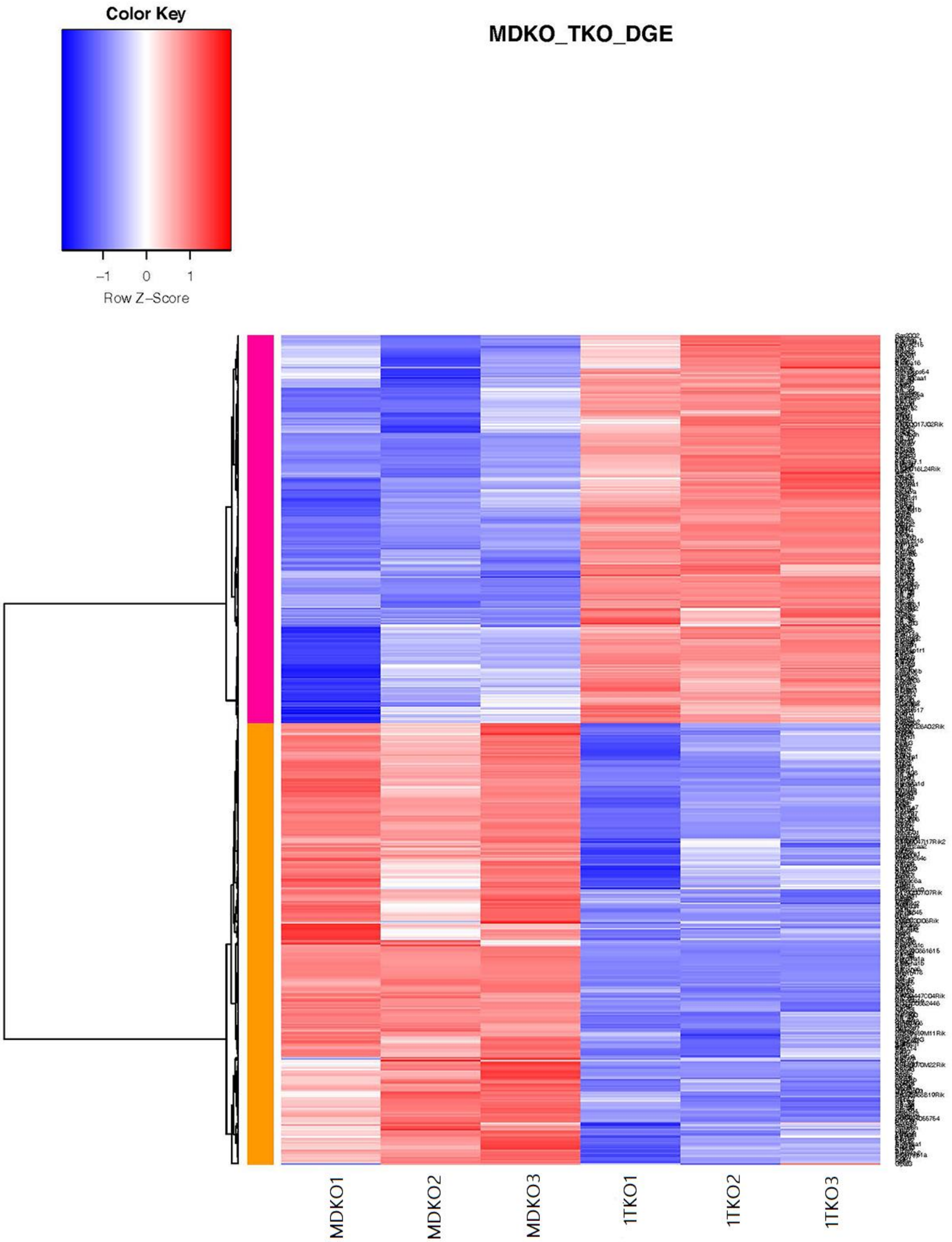

Figure S1. Gene expression profiles between MDKO and TKO hearts. A heatmap shows the differential expression of genes between MDKO and TKO hearts.

A Enriched Components that are down-regualted in MDKO compared to TKO

term description	observed gene count	background gene count	strength	false discovery rate
extracellular region	41	2044	0.46	1.28E-07
extracellular space	29	1131	0.57	2.08E-07
blood microparticle	6	18	1.68	1.19E-06
low-density lipoprotein particle	4	13	1.65	0.00036
plasma lipoprotein particle	5	35	1.31	0.00048
very-low-density lipoprotein particle	4	18	1.51	0.00055
fibrinogen complex	3	7	1.79	0.0011

B Enriched Components that are up-regualted in MDKO compared to TKO

term description	observed gene count	background gene count	strength	false discovery rate
extracellular matrix	28	288	1.04	1.69E-17
extracellular region	60	2044	0.52	1.46E-14
collagen-containing extracellular matrix	18	148	1.13	1.01E-12
collagen trimer	10	78	1.16	4.94E-07
fibrillar collagen trimer	4	9	1.7	0.00027
neuronal cell body	18	688	0.47	0.0026
cell body	19	776	0.44	0.0033
membrane	92	7460	0.14	0.0046
extracellular space	23	1131	0.36	0.0072
cell periphery	61	4443	0.19	0.0072
basement membrane	6	99	0.83	0.009

C Enriched Biological Processes that are down-regualted in MDKO compared to TKO

term description	observed gene count	background gene count	strength	false discovery rate
small molecule metabolic process	33	1489	0.5	8.57E-06
plasma lipoprotein particle remodeling	6	20	1.64	2.84E-05
response to stimulus	77	6616	0.22	7.37E-05
blood coagulation	9	118	1.04	9.85E-05
fibrinolysis	5	15	1.68	9.85E-05
small molecule biosynthetic process	16	481	0.68	9.85E-05
regulation of biological quality	49	3420	0.31	9.85E-05
response to organic substance	40	2553	0.35	0.00012
chemical homeostasis	23	1002	0.52	0.00012
multicellular organismal process	69	5888	0.23	0.00014
response to chemical	49	3532	0.3	0.00014
negative regulation of blood coagulation	6	44	1.29	0.00019
cholesterol efflux	5	25	1.46	0.00022
homeostatic process	28	1494	0.43	0.00022

D Enriched Biological Processes that are up-regualted in MDKO compared to TKO

term description	observed gene count	background gene count	strength	false discovery rate
developmental process	90	5213	0.29	4.22E-08
multicellular organismal process	95	5888	0.26	1.80E-07
extracellular matrix organization	15	180	0.97	1.97E-07
collagen fibril organization	9	40	1.4	1.97E-07
extracellular structure organization	16	214	0.92	1.97E-07
system development	74	4072	0.31	1.97E-07
anatomical structure development	84	4912	0.28	1.97E-07
anatomical structure morphogenesis	48	2089	0.41	4.07E-07
multicellular organism development	78	4603	0.28	7.33E-07
regulation of multicellular organismal process	56	2858	0.34	2.80E-06
response to chemical	64	3532	0.31	2.98E-06

Figure S2. Gene ontology analysis. (**A**,**B**) Enriched Components that are down-regulated (A) or up-regulated (B) in MDKO compared with TKO. (**C**,**D**) Enriched Biological Processes that are down-regulated (C) or up-regulated (D) in MDKO compared with TKO.

Uncropped and unadjusted Western Blot images for Figure 6F

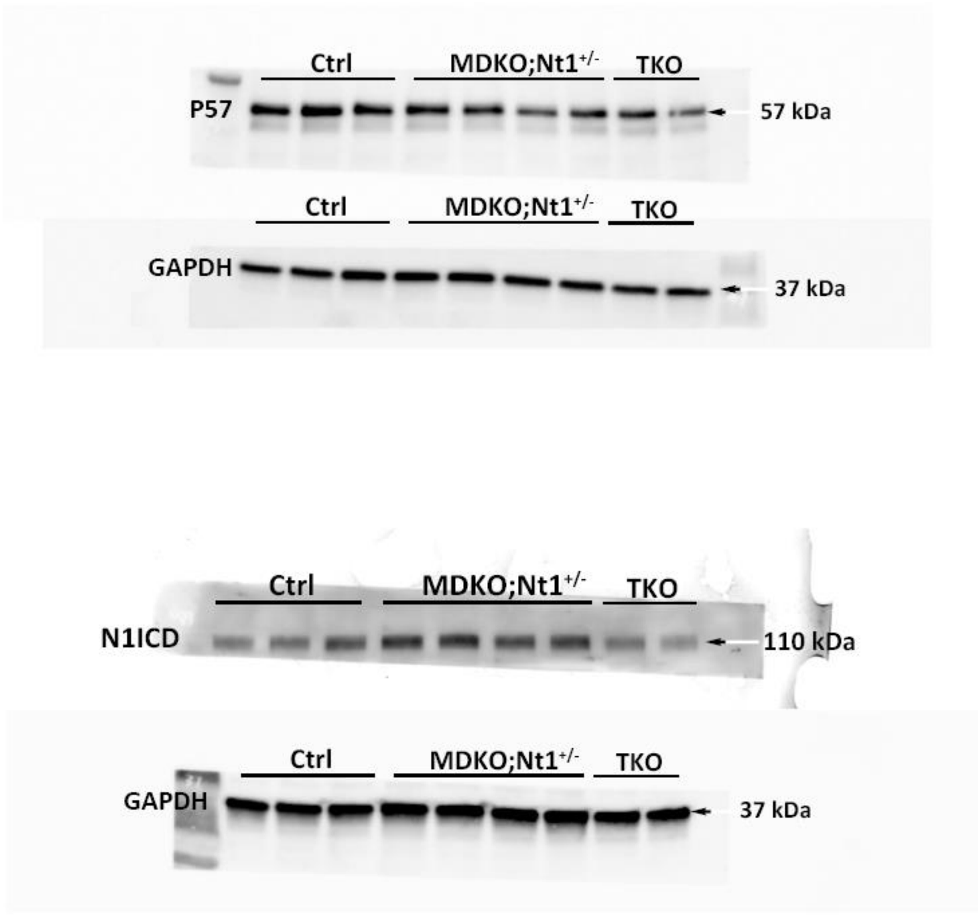

Figure S3. Non-cropped western blots. Non-cropped western blots for Figure 6.

Table S1. qPCR primer list.

Primer Name	Forward	Reverse
Isl1	CCACAAGCAGCCGGAGAAGAC	GAGGGTTGGCGGCATAGCAG
Fgf8	CAGGTCCTGGCCAACAAG	GGTCTCCACAATGAGCTTCG
P57	CAGGACGAGAATCAAGAGCA	GCTTGGCGAAGAAGTCGT

Table S2. mRNA deep sequencing upregulated genes.

symbol	log2FoldChange(TKO/MDKO)	padj
Gm4737	11.96659235	3.02E-21
Gbp4	6.837079842	0.613369247
Nos2	6.252108816	0.69687224
H2-T10	6.108944037	3.84E-13
Zfp108	5.937614272	0.003883676
Cyp26b1	5.912601818	0.725708507
Dnah6	5.851072602	0.003786064
Sh2b2	5.757822713	0.748462301
Ctse	5.716994142	1.89E-39
Pah	5.262018713	1.68E-06
Cbln2	5.203767596	0.027618035
Zfp42	4.923408315	0.011294377
Trpc4	4.844958885	0.38594219
Gm2002	4.837025015	4.86E-10
Aspn	4.76991719	0.017547973
Zfp433	4.705035425	0.739370207
Cd28	4.581314985	0.140338254
Il11ra2	4.57088277	0.318319213
Gm2002	4.57088277	0.318319213
Gm2002	4.57088277	0.318319213
Gm9615	4.53526438	0.204973908
Kcns3	4.503028924	0.507505769
Coq10a	4.497921478	0.777077542
Vmn2r15	4.454816566	0.316352495
Lamtor5	4.359413842	0.572563122
Ptpro	4.256110158	0.811403237
Ugt1a1	4.225349634	0.354552307
Tgm5	4.083461201	0.39339876
Sectm1b	4.047334181	0.57049215
Gm27021	4.036682474	0.401365672
Gm2506	4.009817796	0.849088535
Zfp831	3.97250535	0.437911136
Vmn2r29	3.879579698	0.746654946
Prkcg	3.877494664	0.750827821
Smco3	3.77034883	0.583817905
Gm7168	3.642971556	0.791310175
Ptprq	3.609431315	0.331036007
Rsad2	3.605226957	2.19E-12
Sesn3	3.601747877	0.888776058
Gm6902	3.601747877	0.888776058
Clca2	3.561202203	0.392609964
Scgb3a2	3.557066705	0.679120988
Mc4r	3.556368503	0.796733653
Foxg1	3.532227096	0.000674639
Rlbp1	3.507973307	0.653633021
Cadm2	3.4625154	0.046306864
Musk	3.443849982	0.71149344

Tmem63c	3.441631604	0.825686211
Ascl4	3.439475139	0.577985164
Slitrk1	3.425365411	0.093703589
Fmo2	3.423730987	0.047864005
Ush2a	3.37536252	0.096887922
Fam166b	3.325061876	0.527616985
Olfr1290	3.277040461	0.861161798
Slc22a29	3.238230279	0.708007504
Gsdmc4	3.236159644	0.772731518
Ehf	3.219574059	0.457920921
Ranbp3l	3.207972563	0.785920021
Rasgef1c	3.192359755	0.781933303
Myh3	3.139308516	9.33E-05
Cabp5	3.12279875	0.353956471
Ccl5	3.075776502	0.779298865
Galnt13	3.065187736	0.248742383
Ces1f	3.050429684	0.822452489
Dmrtc1c1	3.034868133	0.690558368
Kcne4	3.028905734	0.115986103
Bcan	3.021794574	0.510672202
Trim30d	3.009719997	0.312782433
Pcdha9	2.990516044	0.376773995
Myt1l	2.98738856	0.665640059
Pianp	2.955816951	0.016774325
Gsdma3	2.913519243	0.696349009
Vmn2r-ps54	2.88717277	0.034646051
Tlr8	2.884792188	0.736075963
Tulp2	2.88411115	0.482832619
Lrfn2	2.829411914	0.697389025
Gm5878	2.826685698	0.698214056
Bhlha9	2.820307138	0.723918967
Rhox4b	2.814753142	0.124375885
Il18	2.810136691	0.822452489
1700030F18Rik	2.808853119	0.765716421
Hpse2	2.794889911	0.757146149
Acot12	2.769666165	0.780009531
Picalm	2.766051079	0.822452489
Vgf	2.75732745	0.766137458
Olfr273	2.723307852	0.748462301
Cilp	2.700439494	0.485391288
Hoxc6	2.686467098	0.21151346
Cldn19	2.662766402	0.846033638
Ace2	2.618907985	0.612132304
Chrnb2	2.618321164	0.878566309
Pde2a	2.607889503	0.400061945
Chodl	2.602833237	0.752548165
Nxph2	2.582811421	0.451576144
Hoxd8	2.579235351	0.261325046
Dsg3	2.54723239	0.782494374
Cxcl15	2.53930312	0.262352438
Tshr	2.535496548	0.212170752
Nkx2-3	2.515791781	0.753143075
Dpt	2.510794088	0.133678845
Lat2	2.497565125	0.707544556
Cfap52	2.452179692	0.524145576
Gm10220	2.449665532	0.875095783
Orm2	2.445107291	0.87295978
Hoxd9	2.43971099	0.76162747

Cpb1	2.438181944	0.888625386
Gabrg3	2.436314499	0.017336963
Gabbr2	2.431846262	0.716445934
Csf3	2.410215804	0.823045603
Gip	2.407922587	0.841296427
Scn1a	2.397124531	0.733861846
Hist2h2aa1	2.382587838	0.049000572
Mymk	2.372810165	0.833219173
Bglap3	2.357127277	3.81E-20
Duox1	2.355491157	0.830356644
Mroh6	2.349980291	0.84041105
Elmod1	2.348132474	0.613369247
Mroh5	2.340141833	0.85377995
Clec4a3	2.331052152	0.754906223
Gm2506	2.313199044	0.736075963
Tssk1	2.30094186	0.590804768
Gvin1	2.288378124	0.053068069
Fam71e1	2.287296429	0.895526424
Mapk10	2.281749057	0.172992214
Serpina3c	2.278905165	0.891960754
Gm1979	2.266263446	0.909802402
Pcdhb9	2.255323411	0.455713737
Cdh16	2.252747052	0.855403705
Sgcz	2.252236267	0.860265596
Otor	2.249061951	0.157298038
Pof1b	2.236799038	0.801161408
Kcnt1	2.219068093	0.867183668
Tnfaip8l3	2.193276039	0.766137458
Cyp2b19	2.190030874	0.893183771
Ankrd7	2.189801706	0.895526424
Otos	2.185520133	0.767897286
Olfr288	2.178611297	0.747401456
Fabp4	2.162726034	0.000134793
Lamp5	2.160754615	0.596388657
Cdh9	2.159435742	0.165141657
Izumo1r	2.155736795	0.858550643
Gria4	2.154610644	0.577985164
Gm5150	2.124289574	0.872608382
Ifit3b	2.123521655	0.779634483
Olfr71	2.122760801	0.658216207
Cyct	2.119350942	0.755153311
B3gnt8	2.115915673	0.878566309
Gbp2b	2.115002984	0.838734386
Fam19a4	2.114921041	0.907144216
Gm18853	2.103545672	0.914032459
Dscaml1	2.096873221	0.933160117
Ccnb1ip1	2.069371491	0.848157323
Cep290	2.064037642	0.899152722
Ms4a4a	2.063294396	0.644687297
Slc9a2	2.061166875	0.800484688
Tnxb	2.057829133	6.65E-12
Kndc1	2.054094379	0.752548165
Cd5l	2.049657969	0.884727454
Dmp1	2.038585233	0.914651623
Gcnt7	2.038306	0.839779475
Apol9b	2.037764755	0.846591495
Gm12248	2.032103319	0.906910553
Eps8l1	2.03016638	6.93E-06

Kcna2	2.010094672	0.776588603
Ptgds	1.995805956	0.690716525
Adcyap1	1.993077557	0.839586495
Efcab8	1.984021748	0.893841411
Pramel5	1.983237866	0.922794899
Chl1	1.977022731	0.692014541
Csprs	1.971912866	0.846591495
Pnoc	1.964776701	0.010327302
Btla	1.959479259	0.846591495
Themis	1.939279699	0.906459923
Etnppl	1.937540108	0.862957291
Slc22a13	1.930529775	0.522535948
Olfr544	1.926210232	0.679399961
Spatc1	1.923593326	0.838734386
Dmrtc1c2	1.918410015	0.924693597
Clec2h	1.915708033	0.60009073
Zic4	1.905408116	0.900499934
Tmem215	1.902983387	0.017091638
Brinp3	1.902607559	0.830356644
Ackr2	1.885648521	0.874544867
Dct	1.8821857	0.436126669
Zfp317	1.87373842	0.955330382
Hbb-b1	1.871816466	2.61E-18
Tusc5	1.864409299	0.91080226
Trim60	1.860029152	0.87427391
Alkal2	1.849162162	0.861820367
Nalcn	1.842458794	0.893841411
Izumo1	1.839937622	0.751774066
Vmn1r61	1.838473419	0.86950172
Prima1	1.831580736	0.308371254
Spint4	1.8285305	0.895526424
Ear1	1.825789299	0.906951294
Ryr1	1.825396715	0.9117512
Cnga2	1.824045867	0.794948446
1700034I23Rik	1.817263406	0.91089661
Lum	1.816996067	8.61E-42
Zscan4d	1.816979379	0.870496004
Pcdhb15	1.813426123	0.615697275
Npy4r	1.81329369	0.752548165
Agtr2	1.797884637	0.224901293
Wdr72	1.780188832	0.749209903
Serinc4	1.774407776	0.543988199
Gm1123	1.773587368	0.899536838
Akain1	1.773013706	0.914830243
C5ar1	1.772703139	0.305707448
Pparg	1.770664257	0.803399016
Gm7361	1.765266608	0.878566309
Fgf7	1.762139554	0.002717708
Rxfp2	1.759324797	0.274724883
Pjvk	1.756638949	0.822079351
Hoxa5	1.751514878	0.0004875
Gm4951	1.745144045	0.852530661
Slpi	1.744821798	0.825803403
Oscar	1.744643826	0.743702176
B3galt2	1.739031827	0.8451558
Trim5	1.739022923	0.18266345
Cd55b	1.73753789	0.769462598
Ptchd1	1.732366605	0.654273769

Cdkl5	1.727685584	0.280701052
C87414	1.724177715	0.777016442
Cfap54	1.721667218	0.212714072
Scn9a	1.721218789	0.798610643
Crct1	1.704073064	0.878566309
Spag6	1.703528662	0.798610643
Olfr1223	1.698935109	0.846591495
Sstr3	1.69253672	0.840516475
Myh8	1.687790615	0.07728559
Impdh2-ps	1.686886243	0.910827448
Deup1	1.686725261	0.846591495
1700010B08Rik	1.685354708	0.9117512
Cpxm2	1.674689946	0.711008272
Cd200r4	1.668828094	0.810687187
Rps15a-ps5	1.667570845	0.912180054
N4bp2l1	1.664812824	0.909061074
Hist1h2bp	1.660829707	0.857932648
Gdf5	1.658088987	0.554325262
Ippk	1.637180074	0.961736907
Dusp15	1.634721028	0.499777037
Mstn	1.632979259	8.92E-10
Pcdha10	1.631889898	0.947839847
Lypd1	1.629985656	0.642561762
Sctr	1.629887952	0.894977531
Clec1a	1.629472022	0.280695365
Kl	1.626741613	0.017547973
Foxb1	1.612286693	0.859699744
Col6a6	1.607042963	3.18E-05
Camsap3	1.603207716	0.507505769
Sele	1.601704193	0.274724883
Alox15	1.601032788	0.921167071
Tchh	1.598498558	0.000221542
Cntn6	1.594577346	0.22068014
Morn3	1.59206764	0.661942005
Nhlrc4	1.591119644	0.517074332
Mgam	1.590836581	0.878566309
Apol8	1.586129222	0.000355154
Sim2	1.586116979	0.831132013
Pcdhb2	1.58433909	0.752548165
Fcgbp	1.583660893	0.930274099
Lox	1.582633629	7.62E-25
Nfasc	1.581986344	0.684719945
Tmem200a	1.579257653	0.876695329
Ascl1	1.576813456	0.909061074
Tmem217	1.575684043	0.906910553
Dazl	1.574959697	0.810554907
Hoxa4	1.574386434	3.95E-05
Acer1	1.572607585	0.878566309
Myog	1.569421185	0.026151023
Il1b	1.563602114	0.838734386
Gm14025	1.563409397	0.78361525
Ogn	1.563047454	3.75E-12
Slc10a6	1.562994793	0.712833755
Abcc12	1.559429685	0.921268579
Icos	1.559269654	0.67038043
C5ar2	1.559157671	0.08078255
Foxs1	1.555853158	0.156589596
Gprc5d	1.552731271	0.907144216

Dnajb7	1.552242689	0.907144216
Hsf4	1.549346355	0.827414822
Marveld3	1.548188486	0.724720605
Gpr31b	1.547924908	0.894977531
Ptprz1	1.54186422	0.796991307
Igll1	1.541674758	0.91618747
Csf2rb2	1.536505827	2.54E-06
Podnl1	1.536007155	0.910827448
Luzp2	1.533495034	0.001837189
Sstr2	1.532888635	0.74019033
Ptpn22	1.532817632	0.543798519
Gpr35	1.532325483	0.183169693
Saa2	1.531014614	0.888625386
P2ry12	1.529880679	0.306078354
Spata21	1.524341178	0.916964849
Adora2b	1.509575475	0.556838937
Npc1l1	1.509244558	0.842328519
Cldn20	1.508639372	0.659468477
Tnn	1.507789606	0.907144216
Tmem150b	1.503682627	0.782154401
Aloxe3	1.503046454	0.552715367
Atp4b	1.502828951	0.91089661
Wfdc18	1.498701487	0.874544867
Btn1a1	1.49606282	0.023410272
1700006E09Rik	1.49361588	0.921268579
Abhd12b	1.492523713	0.452666086
Cox7b2	1.487752811	0.861667381
D930020B18Rik	1.484272433	0.846591495
Fam57b	1.480228112	0.838206592
Serpinb1c	1.479933901	0.888625386
Prelp	1.474412702	0.032392468
Sstr4	1.472038089	0.172992214
Trank1	1.470152284	0.812122767
Zfp385c	1.466040296	0.792006656
Tmprss5	1.457046289	0.779298865
Hsd3b6	1.452808217	3.63E-15
Ackr4	1.448756535	0.944929491
Hoxc5	1.445534083	0.084479441
Bank1	1.445254347	0.878566309
Ddx3y	1.442664291	0.001898708
Scg5	1.442191288	0.039638694
Iigp1	1.436413574	5.85E-17
Asic2	1.434255477	0.697389025
Erc2	1.430691069	0.907144216
Rtp4	1.430155696	0.9117512
Gm17768	1.429563655	0.825686211
Fshr	1.429400445	0.914032459
Olfr1260	1.419382389	0.867374531
Phyhip	1.419020885	0.367725082
Frem3	1.416820896	0.275804076
Eci3	1.415417604	0.777077542
4930562C15Rik	1.409232275	0.360367588
Tnfrsf14	1.405553671	0.40553296
Stra8	1.401150421	0.943898975
Trpa1	1.400156753	0.779746955
Vstm2b	1.399382153	0.923945105
Lif	1.390640731	0.127917796
Hoxd4	1.390403706	0.24727152

Tex13b	1.388488055	0.798610643
Zfp109	1.387690724	0.412332762
Gfap	1.386647031	0.766137458
Nupr1l	1.385852797	0.76162747
Slc37a4	1.381711249	0.920728577
Ifi203	1.380955697	0.633135879
Trpc7	1.380645928	0.810554907
Igsf9b	1.379971768	0.88529255
Pappa2	1.378218693	0.590894753
Susd2	1.37697022	0.007973146
Ecel1	1.376661965	0.888625386
Cdh8	1.376568818	0.001837189
Cckar	1.375353604	0.760580565
Slfn14	1.372125016	2.87E-06
Ccl4	1.371042477	0.914696673
Gm13283	1.365601499	0.867374531
Col6a3	1.365349548	6.02E-16
Asphd1	1.359500771	0.794948446
Acsm2	1.359184606	0.797451786
Mdga2	1.354607975	0.482832619
Gabrq	1.352558654	0.860398399
Sfrp2	1.351149282	9.28E-18
Ccdc85c	1.349769358	0.914778092
Gm3086	1.348397623	0.77898979
Clec3b	1.347939281	0.931547433
Apln	1.346632072	5.37E-06
E130218I03Rik	1.346069691	0.914032459
Tsx	1.345908729	0.782494374
Gng13	1.345257439	0.699389925
Gm14393	1.345090986	0.838734386
Shisa9	1.344079731	0.203415984
Ribc2	1.343356376	0.907346423
Fam46c	1.342031151	1.48E-13
Pkib	1.341724147	0.207742882
Mcoln2	1.340512041	0.74532728
Olfr576	1.338315221	0.86950172
Rs1	1.333604207	0.872391257
Pde1a	1.323637541	1.27E-08
Btbd18	1.320153366	0.828935135
Lhx8	1.319838221	0.911712969
Lvrn	1.316532151	0.850709896
Fam205c	1.311566601	0.907144216
1700001P01Rik	1.309239585	0.82131458
Lonrf3	1.308596545	0.530815297
Mettl21c	1.308373459	0.791310175
Mroh3	1.307219196	0.91853851
Ifi44	1.3049356	0.842041029
Tll2	1.30258222	0.955185819
Sntg1	1.302032764	0.920728577
Fbln5	1.299923093	0.767897286
Dlgap2	1.299150685	0.934597629
Brsk2	1.297081795	0.927639075
Cdkn2a	1.295238311	0.929917747
Rspo4	1.292235235	0.902464467
Eln	1.291919101	2.08E-09
Uba1y	1.288940899	0.858905451
Selenov	1.28876181	0.915888519
Scg2	1.288217132	0.794948446

Aaed1	1.287045232	0.005566495
Btc	1.278328246	0.234791047
Fank1	1.278069229	0.946813528
Pcdha11	1.272961161	0.932922487
Grm7	1.268297353	0.830356644
Ucp3	1.266393998	0.937886548
Ccna1	1.265355109	0.959076092
Mc5r	1.263193739	0.946813528
Tcerg1l	1.260621569	0.249271647
BC049715	1.257308961	0.94430835
Vegfd	1.255306323	0.010040984
Eif2s3y	1.252594805	6.40E-14
LOC102638047	1.252402991	0.925757948
Serpina3i	1.249307716	0.919207859
4931406B18Rik	1.24902659	0.85393171
Npw	1.246823199	0.86950172
Ifi27l2a	1.245719609	0.905209707
Hoxa7	1.231416784	0.909802402
Hist1h2bq	1.229854203	0.739370207
Adamts18	1.227229171	1.63E-08
BC030870	1.225091796	0.898398831
Cdk20	1.224287852	0.731092275
Cfap77	1.223968533	0.596700818
Klk13	1.22051215	0.830356644
Fbxo43	1.219379145	0.921240084
Il7	1.217870684	0.877258953
C1ql4	1.21710339	0.914032459
Zfp114	1.215818592	0.065702652
Gm13306	1.215596733	0.87446373
Zfp92	1.215428407	0.808927921
Esm1	1.211222729	0.063348233
Sln	1.211116382	0.010638745
Csf2rb	1.2079415	8.37E-13
Jaml	1.206964897	0.839076103
Scn4b	1.205911667	1.05E-05
Eqtn	1.204762773	0.734639978
Dtx1	1.201290618	0.761274755
Slitrk2	1.200714696	0.021671016
Tmem45a2	1.199621937	0.916964849
Scd4	1.19500798	0.730154546
Penk	1.194160369	2.68E-05
Hoxb9	1.192425548	0.874544867
Ubtfl1	1.191338472	0.878566309
Rps13	1.191225774	4.70E-06
Ptchd3	1.191059074	0.927969704
H2-T24	1.186713672	0.01468151
Tekt2	1.186495638	0.441427655
Wnt16	1.18564277	0.51178702
Nkx3-1	1.185033556	0.779298865
Usp17la	1.18336757	0.846033638
Nfix	1.182993521	0.000795553
Olfr613	1.180304302	0.051652975
Uty	1.178184614	0.009046236
Il17f	1.176541882	0.577985164
Fam205a2	1.174902477	0.782494374
Fam205a2	1.174902477	0.782494374
Ccdc65	1.172358217	0.78571246
Tm4sf20	1.172351461	0.954876065

Slc10a1	1.168476243	0.895526424
Chad	1.16714808	0.767851575
Tex52	1.16583375	0.679509875
Krt35	1.165408914	0.951345967
Hoxc13	1.165379975	0.951345967
Lrrc32	1.162716483	5.95E-06
LOC108168679	1.158203032	0.133567142
Dbh	1.153097166	0.899733373
Apoo-ps	1.152626369	0.956429004
Gm45951	1.150541216	0.846591495
Tex14	1.150331626	0.909802402
Rd3	1.14970518	0.628441732
Phox2b	1.145628119	0.936551022
Mcemp1	1.138477892	0.946370745
Matn2	1.138223645	1.93E-08
Gramd1b	1.135549992	8.79E-08
Nrtn	1.132041215	0.888776058
Calb1	1.131926398	0.920350374
Patl2	1.128590348	0.857932648
Arsj	1.126067392	0.749196589
Rad21l	1.124324932	0.952518824
C7	1.124219264	0.823045603
Rgs8	1.122335873	0.048944806
Slc1a6	1.119792178	0.399411563
Oas1a	1.114561072	0.796642888
Fndc1	1.111424739	0.335221481
Aldh3a1	1.111304511	0.946370745
Gpr143	1.109021792	0.944034764
Ccdc3	1.105853275	1.11E-07
Ssu2	1.105540175	0.900499934
St8sia4	1.104064355	0.021283277
Siglece	1.103807817	0.861161798
Il6ra	1.103421803	0.811403237
Gabrr2	1.101024044	0.859329905
Ifi207	1.097468231	0.766137458
Btnl9	1.096398798	0.076520901
Bpifc	1.094097737	0.520044817
Irs4	1.093177774	0.23875828
Tspan1	1.092973339	0.941686391
Veph1	1.092315481	0.02352553
Lrrc46	1.091253236	0.679509875
Klhl5	1.089500093	0.933404694
Cct6b	1.08679825	0.888625386
Mgp	1.085222321	0.000574756
Zpld1	1.08277984	0.951025858
Gje1	1.080900685	0.899536838
Prrt1	1.080418741	0.905209707
Foxd1	1.078591604	0.727939791
Nptx1	1.077463722	0.047128848
Ppara	1.07746213	0.394730301
Fkbp6	1.076036825	0.960574622
Kcnj11	1.074512108	0.934597629
Tktl1	1.072065654	0.951792123
Slc9b1	1.070636609	0.874156656
Fcrla	1.069190865	0.955661559
Col1a1	1.067474068	4.63E-18
Impg2	1.065894274	0.38141388
Stpg3	1.061123114	0.951345967

Aqp8	1.060931163	0.207410965
S100a16	1.060642177	0.000795553
Rhbdl2	1.05729569	0.189054586
Syt2	1.055450868	0.711598207
A430078G23Rik	1.054526081	0.888625386
Rbfox1	1.051833122	0.10075876
Mpz	1.051156817	0.914032459
Itga8	1.050815603	0.013899765
Pla2g2f	1.049022625	0.946813528
Cyp4a31	1.0475333	0.951345967
Mybph	1.047158321	0.660268284
Bend4	1.046997272	0.971992168
Akr1d1	1.045826162	0.865509713
Mucl2	1.044721776	0.888625386
Kremen2	1.043536031	0.871449025
Cyp4b1	1.042946264	0.650481231
BC049762	1.042837501	0.920728577
Brinp2	1.042652753	0.722603325
Plcxd3	1.039549103	0.895526424
Nlgn1	1.038941387	0.771776036
Abcc8	1.037922146	0.245996057
Hal	1.03788146	0.782494374
Gpr135	1.035814219	0.20392093
Il23a	1.035094825	0.860398399
Nutm1	1.032032131	0.951345967
Klk14	1.030980328	0.74532728
Pcdhgb2	1.030740584	0.018804433
Gpa33	1.028921607	0.846591495
Msr1	1.028380247	0.482832619
Nav3	1.026652833	0.131767812
Lepr	1.021576177	0.399694125
Tfr2	1.02073547	0.96261644
Trim12a	1.019922146	0.000583271
Nlrp3	1.01725621	0.867374531
Hist2h2ac	1.01667011	0.944034764
Calr3	1.016390691	0.038828771
Pde4c	1.015472155	0.611025461
Lrrtm1	1.013995764	0.66246618
1500015O10Rik	1.012573973	0.472047694
Rprm	1.012245158	0.009756516
Rps3a1	1.009177357	5.94E-05
Nell2	1.008584634	0.330012064
Gabrr1	1.007260886	0.920581873
Lctl	1.005649794	0.951025858
Smoc2	1.004133844	8.97E-16
Hbq1a	1.004001737	0.946813528
Grid2	1.003343009	0.754906223
Adamts16	1.002728269	0.651200829
Pmfbp1	1.000258866	0.865623524
Dao	1.00010725	0.953161751

Table S3. mRNA deep sequencing downregulated genes.

symbol	log2FoldChange(TKO/MDKO)	padj
Alb	-11.60717824	8.27E-20
Afp	-11.08182632	6.25E-69
LOC100862446	-10.58119307	2.08E-29
Eno1b	-10.34082162	7.01E-147
Serpina6	-10.1349939	8.97E-15
Serpina1b	-9.966613117	6.15E-14
Fgb	-9.650021635	3.29E-13
Ambp	-9.340729792	2.36E-12
Fgg	-8.793895674	2.87E-10
Serpina1a	-8.688724349	2.50E-10
Fga	-8.68729614	2.43E-10
Itih2	-7.921881194	1.15E-23
Hnf4a	-7.486320701	1.47E-07
Serpinf2	-7.392551084	3.17E-24
F12	-7.225094362	2.79E-06
Serpina1e	-7.165281318	1.39E-05
Gm11478	-6.883892686	1.99E-31
Kng1	-6.7452209	5.63E-05
Tusc3	-6.599720206	0.643748521
Ugt2b34	-6.500157207	0.000660239
Gjb1	-6.332974697	0.000475507
Proc	-6.296037102	0.000846795
Plg	-6.280563612	1.89E-13
Serpina1d	-6.226580246	0.001089264
Xpot	-6.054541183	0.719531695
Serpina1c	-6.040318291	0.004293364
Itih1	-5.931012385	0.002974254
Apof	-5.913133185	0.009135307
A1cf	-5.714340059	0.007312196
Apoa1	-5.633902767	4.80E-22
Aadac	-5.605231792	0.018554552
Gm5639	-5.526175637	0.224853973
Rhbg	-5.45916567	0.041968276
Serpina10	-5.457147261	0.000360869
Cyp2c68	-5.428914349	0.034105941
Foxa3	-5.427005824	0.018044901
Gm3182	-5.266037588	0.259967542
Aadat	-5.260423966	0.23058357
Sntb2	-5.165722326	0.636482376
Slc30a3	-5.075645961	0.074137712
F7	-5.070244252	0.084479441
Vtn	-5.036569629	7.49E-08
Apba2	-4.938055014	0.423221253
Shbg	-4.763368355	0.022613968
Rhox4d	-4.746933277	0.161772851
Pcdha5	-4.721366343	0.750827821
1810065E05Rik	-4.691621269	0.197464873
Cdhr2	-4.687059233	0.072907267
F2	-4.67684036	1.93E-08
4930555G01Rik	-4.652740436	0.23525871
Gm6756	-4.62573354	0.258809188
Maz	-4.606828964	0.64059001
Gm13090	-4.565068546	0.328991225
Gm3278	-4.506805654	0.585278203
Oxtr	-4.473131984	0.559071287
Zfp853	-4.472837581	0.612132304

Serpind1	-4.446632397	4.44E-10
Otc	-4.427795868	0.275804076
Spaca4	-4.408859322	0.291888095
Hist1h4m	-4.356852579	0.811403237
Creb5	-4.269440106	0.826352864
Mfsd13b	-4.226374706	0.405651384
Tescl	-4.176057851	0.18885586
Izumo4	-4.172865703	0.39537525
Slc2a2	-4.113325355	0.00026657
Matn1	-4.076420135	0.729094498
Afm	-4.022081735	0.055705751
Gm3558	-3.989676508	3.13E-42
P2rx2	-3.976512365	0.73188972
Hist2h2aa2	-3.947433764	0.006247064
Nr1i2	-3.92306762	0.779995153
Lhx3	-3.922504318	0.318319213
Wnt3	-3.92103377	0.335152527
Lrrc7	-3.890984583	0.757146149
Egr4	-3.878069255	0.757146149
AU041133	-3.876384501	0.877258953
Ikzf3	-3.869121159	0.480330426
Bcl2l14	-3.867443945	0.759697592
Hc	-3.862394844	0.276874695
Gstp3	-3.844187554	0.616966653
Gstp3	-3.844187554	0.616966653
Gm3012	-3.835157891	0.766137458
Gm10413	-3.835157891	0.766137458
Ppp3r2	-3.820206447	0.783121989
AF366264	-3.808540582	0.629737944
Spp2	-3.807706706	0.333104318
Abcb11	-3.800505132	0.483553384
Gm8279	-3.76530898	0.23875828
Mmd2	-3.759282556	0.018128635
Prn	-3.737380005	0.888625386
2210409E12Rik	-3.734807584	0.810687187
Gimd1	-3.7032732	0.794396492
Slc13a3	-3.695433951	0.284360442
B9d2	-3.668734197	0.798610643
Pmis2	-3.642532235	0.82957641
Gzmk	-3.640268051	0.814941871
Hgfac	-3.607494857	0.521476734
Chac1	-3.583392183	5.63E-47
Hist1h2ah	-3.545007919	0.903064216
Gm10406	-3.508373844	5.25E-33
Crhbp	-3.501719038	0.466774574
Ptpre	-3.497338915	0.846591495
Gltpd2	-3.405403641	0.487427197
Plekhg3	-3.39333707	0.888625386
Cpb2	-3.391905055	0.330006016
Gm10384	-3.378618833	0.733861846
Chst13	-3.373975126	0.236530799
Lhx5	-3.373267914	0.567425046
Car6	-3.365935917	0.311072828
Apob	-3.335354737	6.42E-09
St8sia3	-3.30001421	0.034008797
Gm3591	-3.295271593	4.54E-66
Cfap73	-3.266777442	0.595778584
Gm6337	-3.246785481	0.713022948

Ltf	-3.245486478	0.733861846
Lipc	-3.235555918	0.640322342
Sh2d6	-3.22354176	0.43634036
Vmn1r53	-3.220221048	0.663665508
Gabrp	-3.216972412	0.408772064
H1foo	-3.177676288	0.75386175
Slc27a5	-3.140569436	0.159734076
Rbp4	-3.126595376	6.60E-20
Fgl1	-3.125769296	2.84E-07
Slc6a12	-3.11761471	0.205079066
Igfbp1	-3.045301806	0.218683832
Krt15	-3.025015623	0.002851981
Amd2	-3.023744535	2.09E-29
Ceacam16	-3.009269461	0.683848217
Pnliprp1	-3.006922654	0.257308553
Klb	-2.999409585	0.031194792
Cwh43	-2.989258591	0.810554907
Pnliprp2	-2.982027688	0.532730591
Fcrl5	-2.968244493	0.795371794
LOC108168187	-2.962169843	0.754906223
Nxnl1	-2.940972389	0.823045603
01-Mar	-2.927804108	0.716445934
Fut1	-2.905098174	0.740475763
Fxyd2	-2.892606847	0.65113588
Ttr	-2.882212313	7.22E-11
Slc7a3	-2.87390917	2.42E-65
Stmn3	-2.871157028	0.333474581
Gm8246	-2.863351717	0.800902393
Rsph6a	-2.852869961	0.770310197
Gpr18	-2.837553484	0.842041029
Speer4a	-2.834077105	0.831132013
Zfp980	-2.829203561	0.269134784
Trpv6	-2.829182514	0.821951721
Apom	-2.825759785	5.82E-08
Pla2g4f	-2.825375643	0.753097677
Hist1h2br	-2.822260103	0.085259806
Slco1c1	-2.812254123	0.405615411
Zbp1	-2.801672655	0.900499934
Gulo	-2.76919165	2.02E-05
Tex35	-2.760658665	0.858169291
Nupr1	-2.756284977	2.91E-10
Ugt2b35	-2.731777382	0.865509713
Apoa2	-2.728130108	6.45E-13
Zfp990	-2.712955923	1.08E-06
Duxbl2	-2.689727101	0.00249341
Tph2	-2.67666569	0.799468308
Cldn18	-2.667045351	0.838220437
Bhmt	-2.657321768	0.020364575
S100a9	-2.64626571	0.044383033
Adh6a	-2.634309825	0.82968756
Spink1	-2.633374692	0.254769768
Prss42	-2.625135148	0.639907402
Edn2	-2.615118019	0.000393562
Grifin	-2.608947292	0.811403237
Entpd8	-2.582776432	0.808927921
Adam3	-2.578698217	0.878566309
Serpina11	-2.575096337	0.811403237
Clrn3	-2.574325897	0.87295978

Ear7	-2.57029558	0.706883966
Masp2	-2.542597884	0.025310153
Ankrd2	-2.535504529	0.70973299
Stfa1	-2.535019152	0.599584656
Slc5a9	-2.525367918	0.616240849
Armc4	-2.521663439	0.874544867
Satl1	-2.514523081	0.877607814
Lilra6	-2.504930139	0.836279268
Vmn2r57	-2.485721458	0.888625386
Ugt1a7c	-2.483726914	0.545920736
Gm5766	-2.480152927	0.921268579
Pilrb1	-2.474175648	0.767885651
Clnk	-2.465733663	0.70973299
Cabp4	-2.46515731	0.70973299
Cabp4	-2.46515731	0.70973299
LOC100861615	-2.451342968	7.63E-39
Qrich2	-2.444888924	0.811403237
Hnf1b	-2.443917689	0.478642428
Mef2b	-2.439288001	0.005603112
Mbd3l2	-2.429186177	0.888625386
Zfp985	-2.416466878	0.001703508
Cphx1	-2.414759902	0.596883837
Apoh	-2.408583929	0.838522465
Ccdc153	-2.398623606	0.855190888
Adm2	-2.393672799	3.78E-09
Kif12	-2.389728694	0.747401456
Bcl2a1d	-2.378994789	0.894506979
Krt13	-2.370172033	0.886035864
Slc39a5	-2.368906139	0.199485753
Ccdc87	-2.36693669	0.895526424
R3hdml	-2.36631284	0.895526424
Ly6g6c	-2.365647156	0.85422039
Ankrd66	-2.3553038	0.871615864
D130040H23Rik	-2.353887095	0.466140281
C1qtnf5	-2.342277289	0.58453945
Ces1d	-2.337408148	0.840507057
Sult6b2	-2.329171592	0.657751054
Fndc11	-2.324977578	0.865646772
Zfp930	-2.320821076	0.425269795
a	-2.317408364	0.86950172
Hoxb1	-2.311316129	0.596926863
Atp6v0d2	-2.304606398	0.811403237
Trib3	-2.298731357	5.45E-26
Cd1d2	-2.293923175	2.75E-05
Chst4	-2.293077234	0.640566693
Arhgap15	-2.287563615	0.400622616
Pax1	-2.28610664	0.396040979
Gys2	-2.278802208	0.795371794
Gdf15	-2.273054805	0.000609421
Prg3	-2.270897477	0.572563122
Rnase6	-2.267976318	0.874156656
Ces5a	-2.264281611	0.852530661
Kynu	-2.262337826	0.867224033
Olfr1420	-2.261101095	0.823045603
Trim66	-2.254985966	2.87E-05
Nrl	-2.236602547	0.846033638
Zfp819	-2.225510896	0.659468477
Lect2	-2.211099859	0.612132304

Prtn3	-2.198745662	0.003883676
Aldh1a7	-2.197362496	1.25E-05
Gpr176	-2.182772085	0.340894122
Gcg	-2.170127137	0.497688315
Otx2	-2.162662895	0.451691331
Habp2	-2.161739379	5.81E-05
Rbp2	-2.160169394	0.465111652
Nlrp1b	-2.156345452	0.729094498
Pgpep1l	-2.154977266	0.776978813
Gjb2	-2.151691188	0.255678166
Gp6	-2.148379136	0.830356644
Doc2a	-2.144523132	0.724115286
Fgf17	-2.141450156	0.846591495
Rec8	-2.141296666	6.04E-14
Mug1	-2.139233232	0.078921267
Apoc2	-2.137608376	0.169808026
Cntnap3	-2.134221623	0.788900343
Sohlh2	-2.130309845	0.851166114
Foxh1	-2.129968949	0.011668594
Zic3	-2.122113592	0.475049515
Fam205a1	-2.118908631	0.896232909
Ceacam2	-2.105472321	0.806635461
Gm3696	-2.09327606	1.90E-23
Morn5	-2.079102456	0.906951294
Pard6a	-2.078558128	0.351052392
Gm7334	-2.073189678	3.57E-20
Sprr1a	-2.067852772	0.01069733
Kcnh6	-2.066446194	0.074637038
Ttll8	-2.063913502	0.839779475
Gbp8	-2.059779414	0.577985164
Cst7	-2.05638694	0.615697275
Cd83	-2.046541586	0.741025848
Lbx1	-2.04067763	0.474629028
3830417A13Rik	-2.038702553	0.60938419
Akr1c12	-2.036236411	0.333350825
4933407L21Rik	-2.033302824	0.302972319
Fbxo15	-2.027798147	0.739420645
Gdf3	-2.024632652	0.524847514
Shisa7	-2.021499099	0.002482059
Nkx6-1	-2.018952903	0.716068151
Muc13	-2.018509966	0.013904296
Sult2b1	-2.012390523	0.865646772
Tmie	-2.003172168	0.524847514
Got1l1	-2.002333171	0.733861846
Clasrp	-1.999460737	0.054997945
2610042L04Rik	-1.997529678	0.172511724
Nlrp5	-1.996057231	0.736341065
Cntn5	-1.9720729	0.827174019
Pfkfb4	-1.966572052	0.357998629
Lrrc66	-1.961059144	0.740950224
Apoc4	-1.960775196	0.509188318
Iqca	-1.955133254	0.896373407
Hus1b	-1.95306022	0.89562551
Cd244	-1.950731656	0.82084991
Mtnr1a	-1.950599641	0.686237892
Aicda	-1.943896921	0.946458387
Tcte3	-1.933046915	0.791617707
Hpx	-1.932714706	0.031288679

Trim80	-1.91628755	0.754906223
Gpr174	-1.909358984	0.922767719
Dapk2	-1.907951895	3.18E-05
Agt	-1.904659161	0.776978813
Tex16	-1.903136669	0.45015764
Mrgprb1	-1.9014121	0.925254287
Kcnb1	-1.900472255	0.525742889
Gdnf	-1.89987947	0.707705125
Il34	-1.898370032	0.755153311
Morc1	-1.890237632	0.870496004
Areg	-1.875369779	0.475301592
Wnt6	-1.871605136	0.226209876
Gm7534	-1.869506534	0.87295978
Stfa3	-1.86872249	0.87295978
4930550C14Rik	-1.866430457	0.907144216
2810459M11Rik	-1.862088349	0.007390004
Pax8	-1.85445329	0.212714072
Asns	-1.852837064	1.30E-21
Rex2	-1.850267013	0.731092275
Bglap	-1.848719549	0.877607814
Nat8f7	-1.847534268	0.946813528
1700028J19Rik	-1.843727626	0.812923815
Slc44a4	-1.842688527	0.712833755
Rp1	-1.841030495	0.946813528
Tex19.1	-1.824960618	0.893841411
Cck	-1.818065493	0.003155613
Krtcap3	-1.811133643	0.899536838
Aldh3b2	-1.807280617	0.894977531
Il17rc	-1.80318487	0.822452489
Tmprss2	-1.802634063	0.686237892
Slc1a7	-1.799117187	0.850687557
Gckr	-1.798388888	0.951025858
Spns3	-1.797727224	0.524847514
Ngp	-1.796589399	0.776978813
Efcab3	-1.793955926	0.727357178
Rgs1	-1.792982865	0.890132829
Sytl3	-1.789643168	0.888625386
Il18bp	-1.771997108	0.716179729
Ovol2	-1.765903761	0.937434786
Cbln1	-1.758445457	0.651200829
Acot3	-1.7583451	0.858915096
Prph	-1.752855335	0.180587087
Sfrp4	-1.752560254	0.877607814
Ccdc110	-1.74108522	0.767851575
Miox	-1.738593597	0.931546053
Gm5796	-1.723830913	7.42E-06
Klk10	-1.712906573	0.867374531
Lhfpl1	-1.711138024	0.006993619
Insl5	-1.710158012	0.902959752
Cd300ld	-1.707328188	0.951345967
Krt4	-1.706972167	0.893841411
Thegl	-1.699357951	0.914032459
Pvalb	-1.698071946	0.297770906
Glra3	-1.691101198	0.867374531
Acp7	-1.689025072	0.835723747
Dynlt1b	-1.687390437	1.05E-11
Cyp26c1	-1.682712011	0.905209707
Pifo	-1.67643969	0.821276622

Disp3	-1.675241294	0.632316286
4932411N23Rik	-1.67291025	0.566747227
Tmem54	-1.672655483	0.85422039
Zmynd12	-1.669409486	0.907346423
Arhgap9	-1.667876993	0.914143956
Klf15	-1.667739868	0.010722472
Olfr1357	-1.662090195	0.921952743
Slc6a2	-1.66106341	0.848015246
Hist1h2ai	-1.660877624	0.924693597
Fcamr	-1.659150808	0.79953967
Zfp872	-1.658045705	0.822452489
Gm1141	-1.656689046	0.588934476
Csmd2	-1.654484267	0.628527414
Gm3629	-1.649665051	0.603272066
Tmem30b	-1.649523631	0.634000607
Nr1h5	-1.646751403	0.038801246
Rnasel	-1.641837856	4.17E-11
Nek10	-1.640510123	0.930274099
Lemd1	-1.638529011	0.806635461
Olfm4	-1.638068547	0.712833755
Gpc5	-1.633771275	0.865646772
Pcdhb8	-1.633342492	0.354552307
Mpo	-1.631380363	1.38E-11
Gm7337	-1.63017911	0.919207859
Egfem1	-1.62764464	0.878566309
Soat2	-1.626920775	7.09E-05
Hist1h3a	-1.623983035	0.838734386
Pkd1l3	-1.62105148	0.698214056
Rtbdn	-1.621008527	0.142876845
Bpifb1	-1.617636434	0.832633287
Il9r	-1.616775058	0.888776058
Pxt1	-1.616605652	0.927639075
C8a	-1.614626012	0.563419467
Gapt	-1.612541656	0.867374531
Slc4a9	-1.611142807	0.843084772
Eomes	-1.600456672	0.554680258
Myh13	-1.594200399	0.721969003
H2-Bl	-1.59223665	0.86950172
C1s1	-1.590300426	0.073109231
2310007B03Rik	-1.588027025	0.924693597
Atf5	-1.585421344	1.31E-26
Cyp4f18	-1.582272394	0.895526424
Hist1h2bg	-1.580709188	0.630176178
Sptbn4	-1.580158412	0.224450034
Mchr1	-1.579094421	0.9117512
Has1	-1.577731186	0.755153311
Fam162b	-1.577259843	0.931547433
8030474K03Rik	-1.570521106	0.622909388
Ccr7	-1.569529246	0.704061013
Epo	-1.567850069	0.909559634
Onecut1	-1.565854395	0.877607814
Rab44	-1.56357257	0.259928325
Cacnb4	-1.559678104	0.197924078
Frmpd4	-1.557479258	0.925548877
Ivl	-1.554979598	0.888625386
Fcmr	-1.554467665	0.795371794
Dnajb13	-1.552585533	0.907144216
Gstm6	-1.551594512	2.31E-06

Kdm4d	-1.549588566	0.916964849
Kiss1r	-1.548746015	0.794948446
Pcp2	-1.547138275	0.953161751
Otop2	-1.543106727	0.946458387
Ceacam12	-1.543014388	0.920452772
Myf5	-1.542200218	0.736075963
Krt79	-1.540082857	0.000755065
Bricd5	-1.539458719	0.90943551
Gm5458	-1.523931755	0.925365809
Acnat1	-1.523823373	0.799275376
Grm1	-1.523344906	0.729094498
Gm6583	-1.52137545	0.865646772
Olfr1258	-1.520640498	0.928028038
Car1	-1.518433269	0.794396492
1700003F12Rik	-1.514842012	0.779999281
Cd200r3	-1.4975218	0.831132013
Npbwr1	-1.497140668	0.850687557
Magi2	-1.496744248	0.961764797
Siglecg	-1.495727626	0.91089661
LOC101055754	-1.486543049	0.000777646
Tlx1	-1.482871031	0.017988597
Ifnz	-1.481553901	0.921268579
Stk33	-1.477499549	0.878566309
Dnali1	-1.474999152	0.779298865
Impg1	-1.474146401	0.884727454
Prss37	-1.473737484	0.774058885
Atp2a1	-1.470211887	0.000865307
Sarm1	-1.470161185	0.576676621
Cyp11b1	-1.46779621	0.924693597
Zfp572	-1.467116828	0.924727078
Psca	-1.466979162	0.906910553
Topaz1	-1.466126567	0.860398399
Hfm1	-1.459917666	0.02259822
Il1rn	-1.457928273	0.9117512
Sprr2a2	-1.456204239	0.842041029
Angptl3	-1.449359287	0.888625386
Dnah5	-1.448991126	0.888625386
Acox2	-1.445895386	0.914032459
Sv2c	-1.444838322	0.129574419
Actn3	-1.439189623	0.000226631
Lgals2	-1.438482389	0.729094498
Sla2	-1.436666028	0.051652975
Tns4	-1.435259156	0.854861451
6030468B19Rik	-1.433916468	0.013102849
Gpr141	-1.432348176	0.924367314
Tppp3	-1.43077204	1.03E-11
Dsg1b	-1.425981169	0.927757308
Pcsk9	-1.425602564	0.006632802
Pnma5	-1.421299668	0.927059165
Eya1	-1.417702169	0.000651209
Sirt5	-1.416404236	0.902682624
Atp6v1c2	-1.415517844	0.830356644
Fuz	-1.412385948	0.911280133
Msln	-1.410333368	5.82E-11
Foxl2	-1.408995986	0.813051429
Pzp	-1.407080477	0.739370207
Amer3	-1.404976306	0.944034764
Cfap44	-1.404132087	0.801161408

Wfikkn1	-1.399496182	0.223478916
Insm1	-1.398199268	0.893841411
Slc22a30	-1.394920365	0.909559634
Slc7a7	-1.392172464	0.962999846
Guca2b	-1.392124352	0.945274783
Rbm44	-1.39105284	0.008987187
Nectin4	-1.3898862	0.19740753
Gm8050	-1.389412862	0.004486494
Hesx1	-1.385905949	0.632390524
Cebpa	-1.383666368	0.782154401
Tekt4	-1.379805196	0.283748308
Gm3383	-1.376295075	0.000457882
Rps7	-1.375863131	1.37E-11
Tspoap1	-1.372460285	0.164829196
Olfr982	-1.372207455	0.879687925
Atp1b4	-1.365434949	0.86950172
Pla2g12b	-1.35905671	0.067487754
Nodal	-1.358900728	0.892750385
Mpig6b	-1.358729687	0.210769591
Cort	-1.358416256	0.755153311
Cth	-1.358192365	3.66E-08
Bco1	-1.357946139	0.783121989
BC100530	-1.355570704	0.773905647
Slco4a1	-1.354007169	0.197924078
Capn11	-1.352454222	0.796733653
Clec4f	-1.351603006	0.920822415
Cdh26	-1.351463061	0.843682085
Hsd17b1	-1.346982622	0.878566309
Pdyn	-1.34393818	0.907346423
Rasal1	-1.342981556	0.838522465
Hist1h2ak	-1.339578516	0.946458387
Cpn1	-1.334064452	0.013235284
Hist1h3f	-1.329219893	0.813047287
Hrk	-1.327971225	0.560923878
Erv3	-1.327644569	0.874156656
Pate3	-1.324360931	0.935610123
Klrk1	-1.322960511	0.91089661
Nphs1	-1.319751619	0.018369189
Faim2	-1.319267872	0.113414229
Gm614	-1.31597234	0.906932838
Akr1c19	-1.313053187	0.330006016
Tdgf1	-1.312911495	0.240200852
Colec10	-1.312728467	0.150605732
Hist1h4b	-1.30881305	0.764135054
Gprin2	-1.308277807	0.851166114
Tex38	-1.306452708	0.811403237
Ccdc73	-1.305918917	0.027038272
Grm6	-1.304030052	0.152747276
Asgr2	-1.30375233	0.895526424
Gm34302	-1.30191854	0.930274099
Kazald1	-1.301513581	0.01839484
Clgn	-1.301395594	0.560923878
Col4a3	-1.301071186	0.515667314
Hist1h1e	-1.30097599	0.734639978
Sftpb	-1.299854579	0.924693597
Prcd	-1.295111252	0.766137458
Hddc3	-1.295020742	1.63E-11
Aox4	-1.294635896	2.34E-06

Ptpn7	-1.294134134	0.000502751
Rasl10a	-1.291224999	0.754906223
Fyb2	-1.28488372	0.573795081
Igdcc3	-1.284318934	9.42E-05
Ugt1a6a	-1.282856298	0.933160117
Slc12a6	-1.281163513	0.899313781
Fgf6	-1.278782706	0.46887346
Tnfsf15	-1.276220972	0.877607814
Ahsg	-1.275674876	0.354552307
Ddit3	-1.274761224	4.21E-10
Gbx2	-1.271767767	0.930354101
Pitx1	-1.270104119	0.746176152
Psg25	-1.269900716	0.937767421
Barx2	-1.269135657	0.12634347
Il15	-1.264050412	0.065083652
Nkx2-6	-1.264000229	0.020676883
Ido1	-1.263525592	0.874452539
4931440F15Rik	-1.262923674	0.800484688
Slc16a8	-1.259236163	0.139756146
Plekhs1	-1.258168525	0.943898975
Fam26d	-1.256730422	0.907673731
Ccdc63	-1.254604517	0.782494374
Fmr1nb	-1.25207393	0.777463784
Tex22	-1.250935895	0.956454218
Serpinb10	-1.249367793	0.951345967
Saa4	-1.248922766	0.888625386
Gpr26	-1.247959554	0.918586318
Dnah11	-1.247240284	0.888625386
Epha10	-1.245579193	0.74532728
Srarp	-1.243374226	0.951345967
Dnah7c	-1.240301224	0.503872483
Best1	-1.237971633	0.766137458
Hist2h3b	-1.237950839	0.910999917
Dupd1	-1.233109819	0.916964849
Tm4sf4	-1.226855252	0.914032459
Nptx2	-1.226742495	0.001721729
E130201H02Rik	-1.226241191	0.951345967
Olfr609	-1.223896356	0.96261644
Hkdc1	-1.221548104	0.049579347
Ripor3	-1.220546906	5.63E-05
Ccl6	-1.220164044	0.766137458
Gm5483	-1.219975041	0.951792123
Wnt7a	-1.214937309	0.648155091
Sgk2	-1.213618963	0.883734136
Asb17	-1.212291273	0.761212129
Adgrg5	-1.212192176	0.785920021
Mei4	-1.209097402	0.401632498
1700014D04Rik	-1.208231245	0.935610123
Cacng5	-1.207077081	0.890917071
a	-1.205078512	3.81E-13
Hist1h4k	-1.204001949	0.874544867
Col22a1	-1.203764035	0.018410489
Fam19a1	-1.203703865	0.946458387
Tspear	-1.202595093	0.904192602
Gdf2	-1.196082708	0.024676473
Vwa5b1	-1.195296174	0.921268579
Ralyl	-1.191057529	0.735085468
Slc14a2	-1.190519558	0.942996034

Abcc6	-1.189571661	0.846033638
Mrln	-1.188840213	0.869320718
Apon	-1.188676948	0.815658332
Ptprn2	-1.18804658	0.716445934
Cfap157	-1.185802306	0.277601192
Phlda2	-1.184090517	0.46612543
Lcn2	-1.183634155	0.94430835
Rgs18	-1.182488109	0.05410606
Panx3	-1.177755547	0.940815971
Glt1d1	-1.176168253	0.841440187
Frmd7	-1.174942271	0.850709896
Serpinb1b	-1.174302944	0.951345967
Palm3	-1.172824424	2.34E-06
LOC108168393	-1.172277118	0.960952634
Ifitm7	-1.171863503	0.874156656
1520401A03Rik	-1.168342661	0.956454218
Trf	-1.167914567	0.0094362
Mthfd2	-1.166602892	8.62E-26
Mc1r	-1.165335445	0.895526424
Hist1h3b	-1.163845594	0.914032459
Bcl3	-1.162989021	0.197637088
Fam43b	-1.162170651	0.937967605
Myb	-1.161196502	0.000770565
Rgs14	-1.161110502	0.716445934
Krt8	-1.1579109	4.21E-10
Carmil2	-1.154532885	0.878566309
Arg1	-1.153960115	0.590324604
Abcg8	-1.15310601	0.838220437
Igfbpl1	-1.152105241	2.10E-11
Cldn23	-1.149924833	0.895526424
Glyatl3	-1.148776579	0.9117512
Crlf1	-1.148531069	0.821036942
Acrv1	-1.146814206	0.878566309
Ly6a	-1.144425535	0.807481654
Myo1g	-1.144157701	0.240200852
Casr	-1.143992916	0.838734386
Odf3l1	-1.141562489	0.830356644
Slc2a5	-1.141522577	0.042260429
Krtap11-1	-1.141156588	0.961736907
0610040J01Rik	-1.140781948	0.76162747
St8sia5	-1.13937416	0.752548165
Il17re	-1.139227108	0.931868305
Sostdc1	-1.139142498	0.249271647
Gjb3	-1.138256783	0.795371794
Ubl4b	-1.135985879	0.952733438
Fstl5	-1.135514224	0.937034774
Hist1h1a	-1.134145409	0.880675827
Mt2	-1.132250261	0.000387968
1700047I17Rik2	-1.130521093	0.006465858
Crym	-1.127568843	0.136033584
S1pr5	-1.125931063	0.615697275
Vmo1	-1.119842227	0.835723747
F10	-1.119294143	0.016927045
Prss22	-1.118589761	0.839779475
Plac9a	-1.117903222	0.956396304
Plekha5	-1.117283509	0.369154686
Cldn4	-1.116720515	0.098653623
Efhc2	-1.113538116	0.755699242

Wdr38	-1.10850842	0.807947444
Oasl1	-1.108057869	0.965079699
Tctex1d1	-1.107871747	0.949897651
Optc	-1.107427269	0.552493812
Prg2	-1.107154815	0.282735662
Tcam1	-1.106145963	0.796733653
Slc16a14	-1.105758063	0.907673731
Igsf1	-1.104923179	0.004429444
Nkx3-2	-1.104502382	0.412451182
Msantd2	-1.104304635	0.003690629
2410137M14Rik	-1.103115551	0.934597629
Tmem52b	-1.103101571	0.94430835
D6Ertd527e	-1.101399342	0.878566309
AI593442	-1.101135267	0.930274099
Ifi27l2b	-1.099965863	0.934597629
Tuba8	-1.099074438	0.67777697
Gdf7	-1.097129796	0.914032459
Shcbp1l	-1.096038431	0.780009531
Lmo3	-1.095565371	0.507513147
Medag	-1.092542679	0.859156797
Pax3	-1.091094819	0.826393044
Fcgr2b	-1.089862236	0.936691465
4930503B20Rik	-1.089669477	0.926447044
Zbtb32	-1.088930617	0.780174202
Gfi1	-1.088917831	0.951792123
Naip6	-1.088665339	0.595265444
Baiap2l1	-1.086772695	1.44E-11
Pklr	-1.086132484	1.40E-08
Osr2	-1.084639278	0.888625386
Pcdhb6	-1.083349322	0.766137458
Slc22a7	-1.082247536	0.732022127
Slc22a8	-1.081893381	0.899246867
Nkain2	-1.076318235	0.912910405
Slc4a11	-1.076022418	0.867374531
Gm2244	-1.075957582	0.895526424
Prss54	-1.07551285	0.929917747
Sod3	-1.072564561	0.870496004
Car4	-1.069393459	7.44E-06
Egr2	-1.068079884	0.815658332
C3	-1.067300346	0.165141657
Psd4	-1.06553211	0.401632498
Pbx4	-1.064336974	0.211894028
Cd3d	-1.063989642	0.369696435
F2rl1	-1.062458693	0.794948446
Hook2	-1.060369412	0.622909388
Gm3488	-1.059665948	0.084944036
Eps8l2	-1.056137078	0.498630831
Jph3	-1.055135573	0.473893384
Erich5	-1.053618061	0.944034764
Efhd1	-1.053459692	0.844511256
Slc26a4	-1.053100425	0.921268579
Spag16	-1.052047229	0.384801795
Il1a	-1.050493328	0.799275376
Slamf1	-1.050173061	0.844939673
Dynlt1a	-1.047781399	1.36E-05
1190007I07Rik	-1.047505844	0.001626581
Epsti1	-1.044839001	0.931486099
Plin4	-1.041356117	0.843340721

Gm4070	-1.040821441	0.942842186
Slc5a11	-1.040312086	0.838734386
Akr1c13	-1.039988179	0.301641333
Mri1	-1.039447041	4.99E-06
Olfr1	-1.037377591	0.86950172
Atp13a4	-1.037333313	0.892750385
Itih3	-1.036135504	0.076210095
Dapl1	-1.035604071	0.923930558
Ooep	-1.032201632	0.502329856
Prmt8	-1.029990417	0.210546561
3110070M22Rik	-1.029548561	0.035336026
Cetn4	-1.028629653	0.515092347
Clec4n	-1.028208408	0.926447044
Exoc3l4	-1.027662458	0.968998483
Gm128	-1.027123369	0.794948446
Ift43	-1.026450079	0.313867902
Tmem59l	-1.026085528	0.888625386
Tnfaip2	-1.025859704	0.010722472
Rbm47	-1.025332941	0.493161659
Mcmdc2	-1.025197456	0.08862693
Olfr1055	-1.022885528	0.956396304
Pou2f2	-1.021988532	0.46612543
Rab9b	-1.021872485	0.780009531
Lipg	-1.021832592	0.000259191
Tbx4	-1.02069112	0.094034766
Hnf1a	-1.020220113	0.912180054
Iyd	-1.01940475	0.804732748
Xlr4a	-1.01722283	0.893841411
Celsr3	-1.017032544	0.900499934
Vmn1r90	-1.015475124	0.808927921
Fam46b	-1.01490541	0.06373078
Large2	-1.013384936	0.158685975
Selenbp2	-1.011770406	0.658514991
Scgb3a1	-1.010067603	0.646524529
Gm14586	-1.009657985	0.951025858
Prss8	-1.009279404	0.878566309
Lyzl4	-1.008309599	0.96261644
1700088E04Rik	-1.007924176	0.053275579
Prss50	-1.007155704	0.961764797
Tnfsf14	-1.007036259	0.860398399
Lhx2	-1.006026461	0.470462153
Nanos3	-1.00537804	0.797451786
Coro2a	-1.004780505	0.45367599
Tnfrsf26	-1.004160297	0.971992168
Taf9	-1.002828979	0.017674991
Slit1	-1.002555349	0.857932648
Baiap2l2	-1.002434505	0.001192021
